# Ranking-Based Salient Object Detection and Depth Prediction for Shallow Depth-of-Field

**DOI:** 10.3390/s21051815

**Published:** 2021-03-05

**Authors:** Ke Xian, Juewen Peng, Chao Zhang, Hao Lu, Zhiguo Cao

**Affiliations:** National Key Laboratory of Science and Technology on Multi-Spectral Information Processing, School of Artificial Intelligence and Automation, Huazhong University of Science and Technology, Wuhan 430074, China; kexian@hust.edu.cn (K.X.); im.pengjw@gmail.com (J.P.); ZhangC_22@hust.edu.cn (C.Z.); hlu@hust.edu.cn (H.L.)

**Keywords:** salient object detection, depth estimation, shallow depth-of-field, ranking loss

## Abstract

Shallow depth-of-field (DoF), focusing on the region of interest by blurring out the rest of the image, is challenging in computer vision and computational photography. It can be achieved either by adjusting the parameters (e.g., aperture and focal length) of a single-lens reflex camera or computational techniques. In this paper, we investigate the latter one, i.e., explore a computational method to render shallow DoF. The previous methods either rely on portrait segmentation or stereo sensing, which can only be applied to portrait photos and require stereo inputs. To address these issues, we study the problem of rendering shallow DoF from an arbitrary image. In particular, we propose a method that consists of a salient object detection (SOD) module, a monocular depth prediction (MDP) module, and a DoF rendering module. The SOD module determines the focal plane, while the MDP module controls the blur degree. Specifically, we introduce a label-guided ranking loss for both salient object detection and depth prediction. For salient object detection, the label-guided ranking loss comprises two terms: (i) heterogeneous ranking loss that encourages the sampled salient pixels to be different from background pixels; (ii) homogeneous ranking loss penalizes the inconsistency of salient pixels or background pixels. For depth prediction, the label-guided ranking loss mainly relies on multilevel structural information, i.e., from low-level edge maps to high-level object instance masks. In addition, we introduce a SOD and depth-aware blur rendering method to generate shallow DoF images. Comprehensive experiments demonstrate the effectiveness of our proposed method.

## 1. Introduction

Breathtaking photography is all about narrative, i.e., the story the image is telling. There are numerous methods to enhance a photo to tell a story, no matter what the subject or the techniques we choose. Shallow depth-of-field (aka shallow DoF), drawing the viewers’ attention to the region of interest by blurring out the rest of the image, is such a technique. With the smartphone being widely used in daily life, we always use the smartphone cameras to capture photos. However, these acquired photos are always all-in-focus due to the narrow baseline and fixed aperture. Hence, more and more attention has been paid to the shallow DoF rendering techniques [1,2,3,4,5] in recent years.

**Figure 1:**
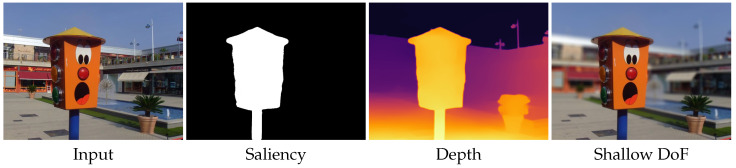
Shallow depth-of-field (DoF) rendering from an arbitrarily captured image.

To render realistic shallow DoF, depth information is required. Some methods use stereo techniques to compute depth maps from stereo images [3,4] and dual-pixel data [2]. However, such methods depend on specific hardware (e.g., stereo cameras or dual-pixel sensors) to capture two views. It is challenging to predict large depth fields due to the narrow baseline. In addition to the stereo-based methods, some other studies [1,2,6] render shallow DoF effects for portrait photos. Although these methods can generate promising shallow DoF images, they cannot generalize well to other scenes. In this paper, we step further to study the problem of rendering shallow DoF effects from an unconstrained image. To this end, we propose a method that consists of a salient object detection (SOD) module for extracting salient objects, a monocular depth prediction (MDP) module for estimating scene depth, and a DoF rendering module for generating shallow DoF images (please refer to Figure 1).

The SOD module indicates the depth range covered by the salient object, in which the focal plane is selected to be within this range. Previous deep learning-based SOD methods [7,8,9,10,11] mainly focus on designing effective network architectures, e.g., HED-based architecture [7], hybrid upsampling operator [8], recurrent localization network [9], pixel-wise contextual attention network [10], and pyramid self-attention module [11]. In order to train these models in an end-to-end manner, the binary cross-entropy loss is widely used. However, such loss, calculated in a pixel-wise manner, cannot explicitly model neighboring relationships. Hence, the predictions often suffer from two problems: (i) the nearby pixels that have different labels (i.e., foreground and background) have the same outputs, named interclass indistinction; (ii) the pixels that share the same label have different outputs, named intraclass inconsistency. To address these issues, an option is to consider structural information. For instance, a fully connected Conditional Random Field (CRF) is always used as a postprocessing strategy to improve spatial coherence [7,12,13]. Inspired by HRWSI (Xian et al., [14]), we propose a novel label-guided ranking loss, which explicitly considers the structure information and can be trained in an end-to-end fashion. Given two points from an image, humans are always good at judging which one is more salient. Thus, a question arises: Can we model this behavior? We sample point pairs from ground-truth saliency maps and annotate which point is more salient for each pair to mimic this process. The sampled pairs can be seen as questions asked by the teacher (i.e., ground truth) and indicate the student (i.e., model) to give answers. Specifically, we define two types of the sampled pairs. One is heterogeneous pair (i.e., the sampled two points are from salient objects and background, respectively), and the other one is homogeneous pair (i.e., cosaliency and cobackground). Given these sampled point pairs, we use a ranking loss [15] to train our model. Unlike pixel-wise losses, the ranking loss only depends on the relative saliency (e.g., point A is more salient than point B or vice versa). More specifically, our label-guided ranking loss is comprised of two terms. One is a heterogeneous ranking loss, which encourages the sampled salient pixels to be different from background pixels. The other is a homogeneous ranking loss that penalizes the inconsistency of salient pixels or background pixels.

The MDP module, which estimates the depth map of an arbitrary image, is used for controlling the degree of blur. Since web stereo depth datasets (e.g., ReDWeb [15] and HRWSI [14]) can only provide depth up to a scale and shift due to the unknown camera baselines and postprocessing, training with pixel-wise losses (e.g., *ℓ*_1_ [5], berHu [16], and scale-invariant loss [17]) cannot generate promising predictions. Therefore, we learn MDP from such pseudodepth data by adopting a structure-guided ranking loss. Different from the loss used in SOD, this ranking loss depends on the depth ordinal relationships. For example, point A is closer than point B or vice versa. In particular, we sample point pairs according to the low-level edge maps and high-level object instance masks, leading to the generation of consistent depth maps with sharp depth discontinuities.

To obtain the final shallow DoF images, we take the all-in-focus image, the saliency map, and the depth map as inputs to the DoF rendering module. The salient map is used to determine the focal plane, while the depth map is used to adjust the blur degree. To synthesize realistic shallow DoF images, we propose a physically motivated method termed scatter-to-gather. Traditional rendering methods always use the gather and scatter operators to render shallow DoF. However, these methods [2,3,18] in practice utilize the layered depth rendering strategy that applies a blur kernel to each depth plane. To keep the refocused plane clear and enable smooth transition around depth discontinuities, our method processes each pixel one by one.

We conduct numerous experiments on SOD, MDP, and shallow DoF rendering. The experimental results demonstrate the effectiveness of our proposed method. In summary, the main contributions of this work are as follows:

- We present an automatic system consisting of a SOD module, an MDP module, and a DoF rendering module for rendering realistic shallow DoF from an arbitrary image.
- We introduce a label-guided ranking loss for SOD. It is a combination of a heterogeneous ranking loss and a homogeneous ranking loss. The heterogeneous ranking loss aims to encourage salient objects to be independent of the background, while the homogeneous ranking loss is dedicated to improving spatial coherence.
- We propose a novel rendering method to render realistic shallow DoF images.

## 2. Related Work

### 2.1. Salient Object Detection

Traditional SOD methods are mainly based on hand-craft features and prior knowledge, including center-surrounding differences [19,20] and boundary prior knowledge [21,22]. Since a detailed survey of these methods is beyond the scope of this paper, we refer the reader to the survey paper [23] for more details. Here, we focus on the reviews of deep learning-based methods.

In recent years, deep learning-based methods [24,25,26] have achieved outstanding performance in visual saliency detection [27,28,29]. For instance, Li et al. [24] design a multilayer fully connected network to predict the saliency score of each superpixel. However, due to a large number of parameters, the fully connected layer decreases computational efficiency. To address this issue, several methods adopt a Fully Convolutional Network (FCN) to generate pixel-wise saliency maps. Liu and Han [25] propose a deep hierarchical salient network to extract both global and local information for SOD. Zhang et al. [8] integrate reformulated dropout layers and hybrid upsampling operations into an encoder-decoder network. To get detail-preserving outputs, multistream networks have been widely used in SOD. Tang and Wu [30] combine cascaded convolutional neural networks (CNN) and adversarial learning for SOD. The two-stream networks, consisting of an encoder-decoder network for global saliency estimation and a deep residual network for local refinement, are designed as a generator. To enable adversarial learning, a discriminator is then incorporated to distinguish the ground-truth saliency maps from the fake ones (i.e., predictions). Recently, contour information [9,31,32] and attention mechanism [10,11,33] have also been attempted for improving the performance of SOD models. Nevertheless, the aforementioned methods focus on network architecture designs, ignoring the explorations of the loss function. The commonly used binary cross-entropy loss, computed in a pixel-wise manner, ignores the neighboring relationships. Training with such a loss suffers from interclass indistinction and interclass inconsistency. To mitigate this issue, we propose a label-guided ranking loss that explicitly models the neighboring relationships. In addition, this operation is similar to the visual attention mechanism of primates (i.e., center-surrounding differences [19,20]).

### 2.2. Monocular Depth Prediction

Deep learning-based MDP algorithms [14,15,16,17] have achieved outstanding performance in recent years. Eigen et al. [17] is the first to apply a multiscale CNN to MDP. Although they use a coarse-to-fine strategy to predict depth maps, the predictions still lack details because of their low resolution. To get finer predictions, some methods [34] train CRF and CNN in a unified framework. Some other methods propose to learn depth by multitask learning, including semantic segmentation [35,36], surface normal estimation [37], and contour detection [35]. However, these methods need additional training labels. Such labels, usually manually annotated, are expensive to collect.

Apart from the aforementioned supervised methods, some researchers attempt to learn depth in a self-supervised fashion [38,39,40]. The basic idea behind these methods is image reconstruction. Instead of using ground-truth depth for supervision, they propose to learn depth or pose in latent space, based on which they can reconstruct the target view. Further, they use the synthesized target view and the ground-truth one to compute the reconstruction loss. Despite the significant progress made, these self-supervised methods still suffer from limitations, such as occlusions, nonrigid motion, and generalization.

The aforementioned methods are mainly trained in constrained scenes and their generalization to other scenes is not well. In other words, these methods trained on one dataset often fail to get promising predictions on a different one. To learn depth in general scenes with a single model, recent studies [15,41,42,43,44] start from constructing in-the-wild RGB-D datasets. For example, Chen et al. [41] propose the DIW dataset, which consists of about 495K natural images. However, they only provide one pair of ordinal relationships for each image, which is not enough to train an accurate MDP model. To address this issue, ReDWeb [15] and MegaDepth [42] were proposed at the same venue. The former comes from web stereo images, while the latter comes from web image sequences. Although these methods have good generalization to unconstrained scenes, their performance can be further improved, especially on depth discontinuities. Thus, Xian et al. [14] propose to guide the network towards the depth discontinuities by low-level edge maps and high-level object instance masks.

### 2.3. DoF Rendering

Realistic DoF rendering usually requires accurate depth information. Thus, some methods use RGB-D images [45,46,47] and stereo image pairs [3,4] to render DoF images. For example, SteReFo [3] interrelates stereo-based depth estimation and refocusing effectively. However, such methods rely on specific hardware, e.g., RGB-D sensors and stereo cameras. Therefore, some other methods [48,49] use off-the-shelf MDP methods to predict scene depth. In addition to explicitly using depth maps to render DOF images, some deep learning-based methods [18,50,51] propose to implicitly learn depth from all-in-focus and shallow DoF image pairs. Specifically, given an all-in-focus image as input, the network is optimized to render a synthetic shallow DOF image. This method, therefore, does not require ground truth supervision on depth. Unlike the aforementioned methods, some other methods [1,6] achieve DoF effects by portrait segmentation. Xu et al. [1] learn a spatially-variant RNN [52] filter to render a shallow DoF image from a portrait photo. Besides, some approaches [2,3,5] which manually select a focal plane, have also been proposed for DoF rendering. By contrast, this paper proposes a method to automatically render a shallow DoF image from an arbitrary natural image.

## 3. Method

**Figure 2:**
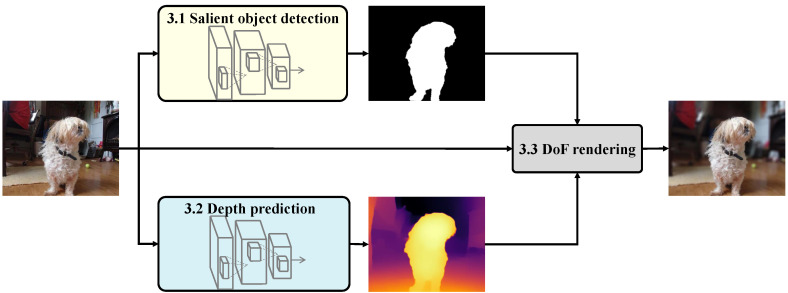
Overview of our proposed method.

In this section, we elaborate on our proposed method for shallow DoF rendering. As shown in Figure 2, our method consists of three modules: salient object detection, depth prediction, and DoF rendering. The rest of this section is organized as follows. Section 3.1 presents a detailed description of our ranking-based SOD module. Section 3.2 describes the ranking-based MDP module. The shallow DoF rendering module is illustrated in Section 3.3.

### 3.1. Salient Object Detection

#### 3.1.1. Label-Guided Ranking Loss

**Figure 3:**
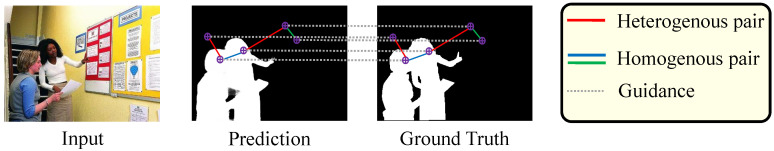
Illustration of our proposed label-guided ranking loss.

Instead of training with a pixel-wise loss, we propose a novel label-guided ranking loss to explore the pair-wise relations in this work explicitly. As shown in Figure 3, the pair-wise relations can be categorized into two groups: (i) heterogeneous pairs, whose labels are contrary (i.e., foreground and background); (ii) homogeneous pairs, whose labels are identical (i.e., foreground and foreground, background and background). To improve interclass distinction and intraclass consistency, our loss function comprises a heterogeneous ranking loss and a homogeneous ranking loss. The basic idea behind the label-guided ranking loss is that we design a heterogeneous ranking loss to encourage the sampled salient pixels to be different from background pixels. Meanwhile, a homogeneous ranking loss is incorporated to penalize the inconsistency of salient pixels or background pixels.

Heterogeneous ranking loss: Given a ground truth saliency map *G*, we randomly sample *N* point pairs (i,j), where *i* and *j* represent the first and second points’ locations, respectively. For each point pair, the first point denoted as $g_{i}$ comes from the background, while the second point belonging to a salient region can be represented by $g_{j}$. Guided by the index (i,j) from ground truth, we use $\left(p_{i},p_{j}\right)$ to represent the sampled point pair from the predicted saliency map *P*. As a result, the sampled set can be represented by *Z*. Note that *N* is image-dependent because different images have different numbers of foreground pixels. We sample N point pairs from each image, where N equals the minimum number of pixels between foreground and background.

To improve interclass distinction, we define the heterogeneous ranking loss as:

(1) $$L_{hete}=\frac{1}{N}\sum_{(p_{i},p_{j})\in Z}\log\left(1+\exp\left(\alpha \left(p_{i}-p_{j}\right)\right)\right)$$

where α is a constant factor, and the term $p_{i}-p_{j}$ can be positive or negative. If this term is positive, which means $p_{i}$ has a greater possibility to be foreground, the loss $L_{hete}$ would be large. In order to minimize the $L_{hete}$, the term $p_{i}-p_{j}$ should be as small as possible. Therefore, this loss encourages the predicted $p_{i}$ and $p_{j}$ to be background and foreground, respectively. Meanwhile, it enlarges the difference between $p_{i}$ and $p_{j}$.

Homogeneous ranking loss: $L_{hete}$ only measures the difference between salient objects and background, which ignores the intraclass consistency. Therefore, we supplement a homogeneous ranking loss that minimizes the intraclass difference. Instead of using a pixel-wise MSE loss, the homogeneous ranking loss explores the pair-wise relations in an explicit way. Considering that there exist two relations of the homogeneous pairs (cosaliency and cobackground) and the scales of losses are different, the homogeneous ranking loss is thus comprised of ${\tilde{L}}_{co-bg}$ and ${\tilde{L}}_{co-sal}$.

To be specific, We define the pixels sampled from background as $Z_{b}=\left\{p_{i}|i=1,\ldots ,N\right\}$ and the pixels sampled from salient objects as $Z_{s}=\left\{p_{j}|j=1,\ldots ,N\right\}$. Then we permute $Z_{b}$ and $Z_{s}$ to get $\hat{Z_{b}}$ and $\hat{Z_{s}}$. So the losses of cobackground pairs and cosaliency pairs can be calculated by:

(2) $${\tilde{L}}_{co-bg}=\frac{1}{N}\sum_{p_{i}\in Z_{b},\hat{p_{i}}\in \hat{Z_{b}}}{\left(p_{i}-\hat{p_{i}}\right)}^{2}$$

(3) $${\tilde{L}}_{co-sal}=\frac{1}{N}\sum_{p_{j}\in Z_{s},\hat{p_{j}}\in \hat{Z_{s}}}{\left(p_{j}-\hat{p_{j}}\right)}^{2}$$

where ${\tilde{L}}_{co-bg}$ and ${\tilde{L}}_{co-sal}$ measure the consistency of background and salient objects, respectively. During training, we observe ${\tilde{L}}_{co-sal}$ is ten times larger than ${\tilde{L}}_{co-bg}$. As a result, we use a hyperparameter σ to balance the difference. Thus, the homogeneous ranking loss can be formulated as:

(4) $$L_{homo}={\tilde{L}}_{co-bg}+\sigma {\tilde{L}}_{co-sal}$$

Finally, we define the label-guided ranking loss as:

(5) $$L_{sal}=L_{hete}+\lambda L_{homo}$$

where λ is a balancing factor. The whole computational procedure of the label-guided ranking loss is summarized in Algorithm 1.

**Algorithm 1** The procedure for label-guided ranking loss**Input:** Ground truth saliency maps *G*, predictions *P***Output:** Label-guided ranking loss *L* 1: Guided by salient objects from *G*, sample pixels $Z_{s}$ from *P* 2: Guided by background from *G*, sample pixels $Z_{b}$ from *P* 3: Permute $Z_{s}$ and $Z_{b}$ to get $\hat{Z_{b}}$ and $\hat{Z_{s}}$ 4: Compute heterogeneous ranking loss according Equation (1) 5: Compute homogeneous ranking loss according Equation (4) 6: Output final loss $L_{sal}$ according Equation (5)

#### 3.1.2. Network Architecture

**Figure 4:**
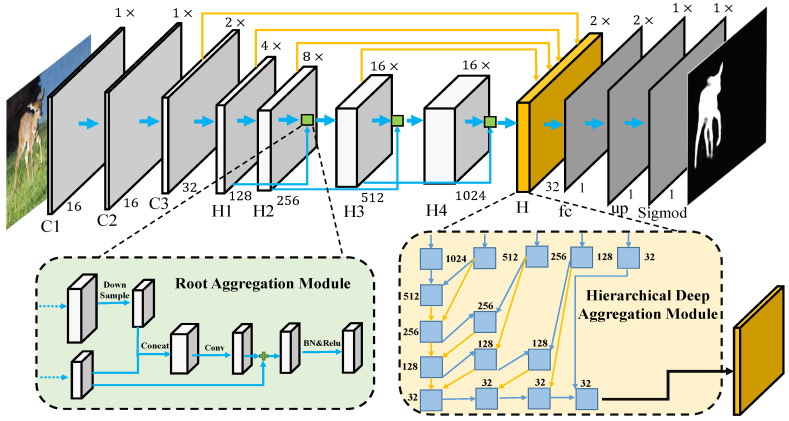
Illustrations of the proposed network architecture. In the hierarchical deep aggregation module, the orange lines indicate deconvolutional operators, and the blue blocks consist of convolution/BN/ReLU layers.

Figure 4 illustrates the schematic representation of our encoder-decoder network architecture. The whole network is based on the deep layer aggregation network structure [53], and we utilize the DLA-60 as our backbone network. In the *encoding* part, we adopt 3 convolution blocks (C1, C2, C3) and 4 hierarchical deep aggregation modules (H1, H2, H3, H4). Specifically, we set the convolutional kernel size to 7×7 in C1 and 3×3 in C1, H1, H2, H3, and H4. As shown in Figure 4, we maintain feature maps in C1 and C2 at the same resolution as the input image, and then the feature maps are downsampled via a convolution layer with a stride of 2. The hierarchical deep aggregation modules H1, H2, H3, and H4 have {1, 2, 4, 1} stages. For each stage, it contains two residual blocks and an aggregation node. The aggregation node, used to combine and compress its inputs, can be based on any block or layer. For simplicity and efficiency, we use a single 3×3 convolution followed by batch normalization and nonlinear activation. Besides, we use skip connections and a root aggregation node to fuse the feature maps between two continuous hierarchical deep aggregation modules. For example, as shown in Figure 4, the root aggregation module combines the features generated by H1 and H2. In particular, we use a 3×3 convolution layer with a stride of 2 to downsample the feature maps from H1, then concatenate with the feature maps from H2 followed by a residual block. To expand receptive fields without losing resolution, we utilize dilated convolution in the last hierarchical deep aggregation module.

In the *decoding* part, we adopt a hierarchical deep aggregation module (H), a convolution layer (FC layer), a deconvolution layer (UP layer), and a sigmoid layer. As shown in Figure 4, we utilize the feature maps generated from (C3, H1, H2, H3, H4) in the encoding part. The whole hierarchical deep aggregation module, successively aggregating and upsampling feature maps, contains four levels. For example, at the first level of H, we fuse H4 and H3 to obtain feature maps l1 that have the same dimension as H3. At the following level, we fuse the l1 and H3 to construct $h2_{1}$, which has the same dimension as H2. Similarly, we combine the $h2_{1}$ and H2 to obtain feature maps with the same dimension as H2. Given an input image at resolution 256×256×3, the hierarchical deep aggregation module generates the final feature maps at resolution 128×128×32. To get our final output (256×256×1), we stack a FC layer (1×1 kernel size), a deconvolution layer and a sigmoid layer.

The traits of our network are twofold. Firstly, in contrast to most prior works [9,10] that only aggregates features from neighboring layers, our network instead leverages the information of most former layers via skip connections, thus integrating information at different levels. Secondly, the parameters of our model have been greatly reduced via deep layer aggregation, which enables fast salient object detection.

### 3.2. Monocular Depth Prediction

To render realistic shallow DoF effects from an arbitrary image, depth information is required. As recent MDP methods commonly use the network architecture proposed by Xian et al. [15], we also use the same one to predict depth maps. The network is mainly comprised of an encoding backbone and a multiresolution fusion module. The encoding backbone extracts features of different resolutions and semantics. The multiresolution fusion module fuses coarse high-level semantic features with fine-grained low-level features, which enables high-resolution outputs and preserves fine details simultaneously. Since the MDP module is not a contribution of this paper, please refer to the reference [15] for more details.

We train the MDP model on HRWSI [14] dataset that consists of 20K high-quality training images. These data have unknown depth scale and shift factors, directly using pixel-wise losses (e.g., $\ell_{1}$, $\ell_{2}$, and scale-invariant loss) cannot get promising predictions [14]. Therefore, we use a ranking-based loss for training. Given a RGB image *I*, we learn a function D=F(I) in a supervised manner, where $D\in R^{h\times w\times 1}$ is the generated depth map. The loss can be computed on a set of point pairs with ordinal annotations. In particular, for each point pair with predicted depth values $\left[d_{0},d_{1}\right]$, the pair-wise ranking loss can be formulated as:

(6) $$\chi \left(d_{0},d_{1}\right)=\left\{\begin{array}{cc} \log(1+\exp\left(-\kappa \left(d_{0}-d_{1}\right)\right)), & \kappa \neq 0\\ {\left(d_{0}-d_{1}\right)}^{2}, & \kappa =0, \end{array}\right.$$

where κ is the ground truth ordinal label, which can be derived from a ground truth depth map:

(7) $$\kappa =\left\{\begin{array}{cc} +1, & d_{0}^{*}/d_{1}^{*}\geq 1+\sigma ,\\ -1, & d_{0}^{*}/d_{1}^{*}\leq \frac{1}{1+\sigma },\\ 0, & otherwise. \end{array}\right.$$

Here, σ is a tolerance threshold [14] that is set to 0.03 in experiments, and $d_{i}^{*}$ represents the ground truth depth value. This loss encourages the predicted $d_{0}$ and $d_{1}$ to be the same when the point pair are close in the depth space, i.e., $\kappa_{i}=0$; otherwise, it would enlarge the difference between $d_{0}$ and $d_{1}$ for minimization. Then, the ranking loss of the sampled pairs can be computed by:

(8) $$L_{rank}=\frac{1}{N}\sum_{i}\chi (d_{i,0}-d_{i,1}).$$

where *N* is the number of sampled pairs. Instead of random sampling, we follow HRWSI [14] to combine low-level edge-guided sampling and high-level object instance sampling. In this way, the networks would pay attention to the salient structure of the given image.

To encourage smoother gradient changes and sharper depth discontinuities in the predicted depth maps, we add a multiscale scale-invariant gradient matching loss. Given $R_{i}=D_{i}-D_{i}^{*}$, this loss can be defined as:

(9) $$L_{grad}=\frac{1}{M}\sum_{s}\sum_{i}\left(\right|\nabla_{x}{R_{i}}^{s}\left|+\right|\nabla_{y}{R_{i})}^{s}\left|\right),$$

Here, *M* and $R^{s}$ represent the number of valid pixels and the difference of depth maps at scale *s*, respectively. In our experiments, we use four scales.

By combining the ranking loss $L_{rank}$ and the multiscale scale-invariant gradient matching loss $L_{grad}$, the final loss for training the MDP model is:

(10) $$L_{depth}=L_{rank}+\beta L_{grad},$$

Following [14], we set the β to 0.2 in our experiments.

### 3.3. Shallow DoF

To render realistic shallow DoF, we design a physically motivated method termed “scatter-to-gather” (S2G). The basic idea, that the light scattering can be converted to a gathering operation indirectly, is similar to [2]. However, we process each pixel one by one instead of using the layered depth rendering strategy. Given an input image $I_{a}$ and a blur kernel *K*, the rendered DoF image *B* can be computed by:

(11) $$B=\sum_{\Delta i}I_{a}\left(i+\Delta i\right)K_{i+\Delta i}\left(-\Delta i\right),$$

Typically, the point spread function shape is circular. We thus use the disk blur kernel to synthesize realistic shallow DoF images. According to [2], the radius of kernel can be computed by:

(12) $$r=Lf|D\left(p_{i}\right)-d_{f}|,$$

Here, *L* is the aperture size, *f* is the focal length, $D(p_{i})$ is the inverse depth of pixel $p_{i}$, and $d_{f}$ is the depth of focus. Since the aperture size and focal length belong to camera factors, we use *A* to represent the multiplication of *L* and *f*. Note that, *A* controls the maximum blur degree.

As illustrated in Algorithm 2, we start from an all-in-focus image $I_{a}$ with its predicted saliency map *S*, normalized depth map *D*, and camera factor *A*. Note that we view the depth map *D* as an inverse depth map during the whole rendering process. We first calculate the depth of focus $d_{f}$ by computing the median of the depth range covered by the salient object. For each pixel $p_{i}$ in an all-in-focus image, we use two accumulated terms $w_{sum}$ and $c_{sum}$ to record its weight and color intensity, respectively. Then, we find the neighboring pixels of $p_{i}$ according to the maximum blurring radius *r*. If the blur radius *r* is larger than the distance *l* between two pixels, the pixel $p_{j}$ will cast to the pixel $p_{i}$, and the weight will be divided by the square of *r*. After traversing all the neighboring pixels of pixel $p_{i}$, we can get the color values at the location $p_{i}$ by dividing the accumulated weight.

**Algorithm 2** The pipeline of rendering method S2G**Input:** all-in-focus image $I_{a}$, saliency map *S*, depth map *D*, camera factor *A***Output:** DoF image $I_{d}$ 1: $d_{f}\leftarrow \mathrm{CalDepthFocus}\left(D,S\right)$ 2: **for**
$p_{i}\leftarrow \mathrm{TraverseImage}\left(I_{a}\right)$
**do** 3: $w_{sum}\leftarrow 0$ 4: $c_{sum}\leftarrow 0$ 5: **for**
$p_{j}\leftarrow \mathrm{FindNeighbor}\left(p_{i}\right)$
**do** 6:  $l\leftarrow \mathrm{Dist}(p_{i},p_{j})$ 7:  $r\leftarrow A\cdot |D\left(p_{j}\right)-d_{f}|$ 8:  $w\leftarrow 𝟙\left(r-l>0\right)\cdot \frac{1}{r^{2}}$ 9:  $w_{sum}\leftarrow w_{sum}+w$ 10:  $c_{sum}\leftarrow c_{sum}+w\cdot I_{a}\left(p_{j}\right)$ 11: **end for** 12: $I_{d}\left(p_{i}\right)\leftarrow \frac{c_{sum}}{w_{sum}}$ 13: **end for**

## 4. Experiments

### 4.1. Salient Object Detection

Following [9,10,54], we use the DUTS-TR dataset [55] for training. We resize images to 256×256 with random horizontal/vertical flipping to avoid overfitting during training. We train our model using stochastic gradient descent (SGD) with an initial learning rate of 0.1, which is decayed by ×0.1 every 15 epochs. The momentum and the weight decay is set to 0.9 and 0.0005, respectively. The whole network is trained for 40 epochs with batch size 84 on two NVIDIA GTX 1080TI GPUs. We use the proposed label-guided ranking loss to train our network and set α, σ, and λ to 3.0, 0.1, and 1.0 in our experiments.

To evaluate the performance of our salient object detection module, we compare our method with the state-of-the-art approaches on six widely used saliency datasets: SOD [56], ECSSD [57], PASCAL-S [58], HKU-IS [24], DUT-OMRON [59] and DUTS [55]. SOD contains 300 testing images, which are generated from the Berkeley segmentation dataset. Most images in this dataset have multiscale salient objects and complex backgrounds. ECSSD has 1000 images with various natural scenarios. PASCAL-S dataset contains 850 natural images, which are generated from the PASCAL VOC 2010 segmentation dataset. HKU-IS includes 4447 images that have multiple salient objects with low color contrast and various locations. DUT-OMRON contains 5168 challenging images that have one or more salient objects. DUTS is the largest salient object detection benchmark dataset. It consists of 10,533 training images (DUTS-TR) and 5019 testing images (DUTS-TE).

#### 4.1.1. Ablation Studies

**Comparison with baseline:** The label-guided ranking loss consists of two terms: the heterogeneous ranking term and the homogeneous ranking term. To analyze each part’s contributions, we explore various configurations and evaluate the models on six datasets. We report maximum $F_{\beta }$-Measure, MAE, and structure-measure in Table 1. $L_{hete}$ means that we train the model with the heterogeneous ranking loss that only computes heterogeneous pairs’ losses. $L_{hete}+{\tilde{L}}_{co-sal}$ is comprised of the heterogeneous ranking loss and the homogeneous ranking loss on cosaliency pairs. Note that the ${\tilde{L}}_{co-sal}$ term only computes the losses of pairs sampled from salient objects. Similarly, the $L_{hete}+{\tilde{L}}_{co-bg}$ term consists of the heterogeneous ranking loss and the homogeneous ranking loss on cobackground pairs. We use $L_{hete}+L_{homo}$ to represent the proposed label-guided ranking loss. As shown in Table 1, one can observe that adding ${\tilde{L}}_{co-sal}$ improves the performance when compared to $L_{hete}$. However, the improvements are limited, which means only considering co-salient pairs is not enough. In addition, we explore the combination of $L_{hete}$ and ${\tilde{L}}_{co-bg}$, which further improves the performance. Furthermore, we incorporate the $L_{hete}$ and $L_{homo}$ together to predict more accurate saliency maps.

**Figure 5:**
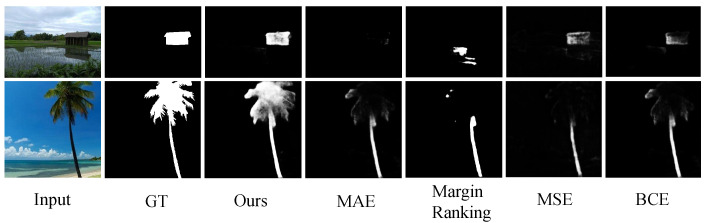
Qualitative results obtained by using different loss functions. Our results are more visually consistent with the ground-truth maps.

**Comparison with other losses:** To demonstrate the effectiveness of our loss, we train the same network architecture with different loss functions. In particular, we compare our loss with four losses (Margin Ranking, MAE, MSE, and BCE). Table 2 shows the maximum $F_{\beta }$-Measure, MAE, and structure-measure scores on six challenging datasets. In addition to quantitative evaluations, we also show some qualitative examples in Figure 5. One can observe that our label-guided ranking loss achieves the best performance. Although we compute losses only on a sparse set of point pairs, the quantitative and qualitative results demonstrate that our model still performs better than those trained with dense per-pixel losses.

**Figure 6:**
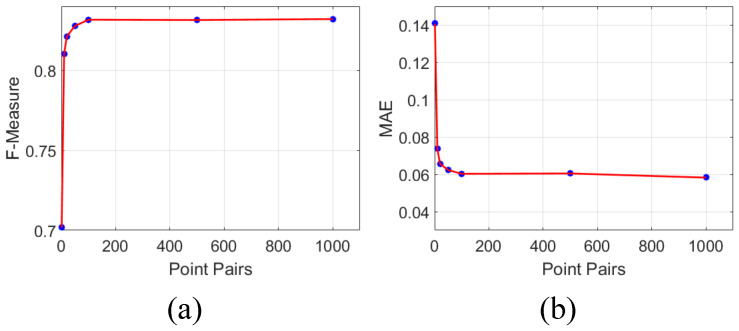
Ablation experiments on the amounts of sampled point pairs. Specifically, we sample {1, 10, 20, 50, 100, 200, 500, 1000} point pairs per image. (**a**) F-measure; (**b**) MAE.

**Impact of the amounts of point pairs:** To analyze the impact of the amounts of point pairs, we sample a different number of pairs during training on DUTS-TR, and evaluate these models on DUTS-TE. Figure 6 shows the maximum $F_{\beta }$-Measure and MAE scores when trained with a different number of pairs. One can observe that training with more pairs improves performance. As the label-guided ranking loss uses an online sampling strategy, the diversity of samples would not be a key factor as the number of iterations increases. Besides, we did not see a significant difference in time consumption.

#### 4.1.2. Comparison with State-of-the-Arts

We compare our method against other 12 state-of-the-art algorithms, i.e., KSR [60], DCL [12], UCF [8], GBR [31], SRM [61], Amulet [62], DSS [7], DGRL [9], BdMPM [54], PiCANet [10], RAS [33], and MLMSNet [32] on six public datasets.

**Figure 7:**
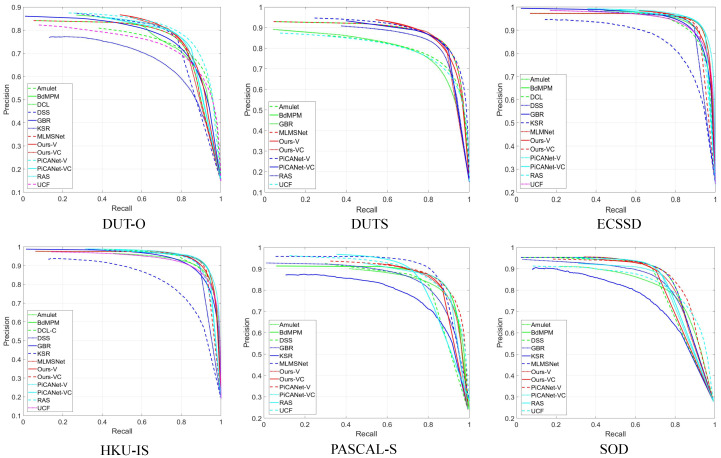
Comparison on six datasets in terms of the PR curve.

**Figure 8:**
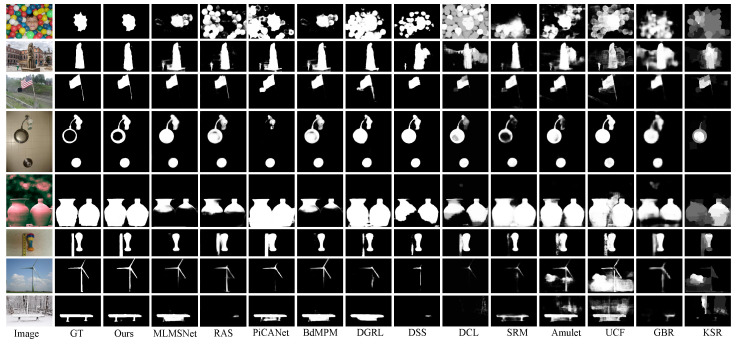
Qualitative comparison of saliency maps. We compare our model with MLMSNet [32], RAS [33], PiCANet [10], BdMPM [54], DGRL [9], DSS [7], DCL [12], SRM [61], Amulet [62], UCF [8], GBR [31], and KSR [60].

**Quantitative and qualitative results:**Table 3 shows the quantitative comparison in terms of maximum $F_{\beta }$-Measure, MAE, and structure-measure. For a fair comparison, we also use VGG-16 and Resnet-50 as our backbone model. Since DSS [7], DCL [12], and PiCANet [10] use CRF [63] to refine their predictions, we use CRF to refine saliency maps as well. The PR curves on six datasets are given in Figure 7. As shown in Table 3, our models achieve competitive or better performance when compared to other state-of-the-art methods. In Figure 8, we further show qualitative comparisons of our method against other methods. One can observe that our method can predict more accurate saliency maps that coincide with the ground truth masks. More specifically, our method can tell apart two salient object instances with similar appearances (e.g., the 5th and 6th rows) and preserve the structural consistency of a salient object (e.g., the 3th and 7th rows ). However, other methods suffer from the two problems (i.e., interclass indistinction and intraclass inconsistency), which holds our basic idea.

### 4.2. Monocular Depth Prediction

Our MDP model, based on a ResNet101-based encoder-decoder architecture [15], is trained on the HRWSI dataset [14]. In order to evaluate the performance of the MDP module, we compare it against other methods on six RGB-D datasets, including NYUDv2 [64], Ibims [65], TUM [66], KITTI [67], Sintel [68], and DIODE [69]. Note that these datasets were unseen during training. The NYUDv2 dataset, consisting of 654 indoor RGB-D image pairs, is captured by a Kinect depth sensor in indoor scenes. Ibims is a high-quality RGB-D dataset specially designed for testing MDP methods. It contains 100 indoor RGB-D pairs with a deficient noise level, sharp depth transitions, no occlusions, and high depth ranges. TUM is also an indoor RGB-D dataset, which mainly focuses on moving people. Particularly, there are 11 image sequences with 1815 images for testing. In addition to testing on indoor scenes, we also test methods on outdoor datasets. KITTI is the widely used outdoor dataset used for testing MDP methods. In our experiments, we use the split (697 images) provided by Eigen et al. [17] for evaluation. Moreover, we also evaluate methods on Sintel, a synthetic RGB-D dataset with accurate ground truth depth maps. This dataset is comprised of 1064 images derived from an open-source 3D animated film. Additionally, we test MDP methods on the official test set (771 images) of DIODE, which contains both indoor and outdoor scenes.

**Quantitative and qualitative results:** In Table 4, we compare our MDP model with 7 state-of-the-art methods, including DIW [41], DL [5], RW [15], MD [42], Y3D [44], MC [43], and HRWSI [14]. For the definition of the metrics, please refer to [14]. As shown in Table 4, one can observe that our MDP model outperforms other methods, exhibiting good generalization performance. Despite being trained with less data when compared to DIW, MD, Y3D, and MC, our MDP model still exhibits better generalization performance. The reasons may lie in the quality of training data as well as the structure-guided ranking loss. The HRWSI dataset has diverse training samples with high-quality ground truth depth data. The structure-guided ranking loss guides the model toward the regions that better characterize the structure of the image.

**Figure 9:**
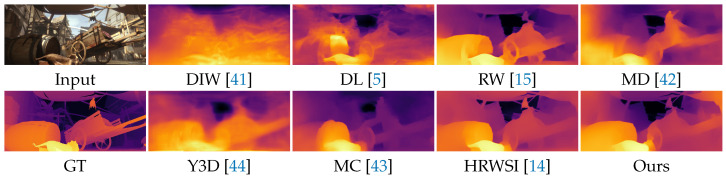
Qualitative results of different MDP methods.

We further show some qualitative comparisons in Figure 9. Our MDP model can get more accurate predictions, which has more consistent depth with sharper depth discontinuities.

### 4.3. Shallow DoF

We conduct experiments on 4D Light Field (4DLF) dataset [70], that consists of 20 photorealistic scenes. For each scene, it provides an all-in-focus image, a disparity map, as well as 9×9 light fields. We use the light field refocusing method [71] to synthesize the DoF images as ground truth. In particular, each image is refocused at five disparity planes, i.e., −1.5 px, −0.75 px, 0 px, 0.75 px, and 1.5 px. To verify the effectiveness of our DoF rendering module, we implement two DoF rendering methods (i.e., RVR [18] and SteReFo [3]).

**Figure 10:**
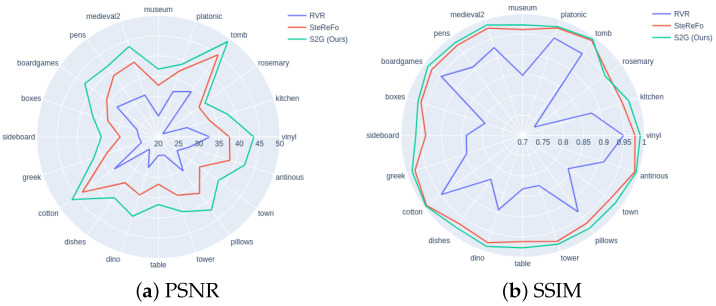
The radar charts summarize the scores on different scenes.

**Figure 11:**
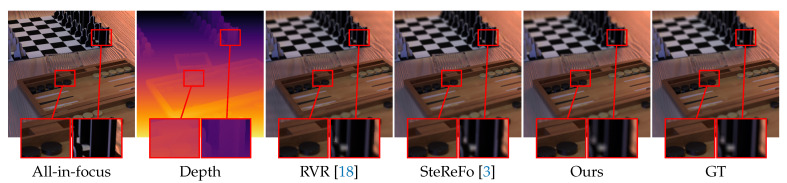
Qualitative results of different DoF rendering methods on the 4DLF dataset. Best viewed zoomed in on-screen.

Table 5 reports the quantitative results of these methods in terms of PSNR and SSIM, and Figure 10 summarizes all scores computed on the three methods and 20 scenes. One can observe that our proposed method outperforms other methods by a large margin. In Figure 11, we further show the qualitative results of these methods. Given an all-in-focus image, these DoF images are generated by focusing on the plane of disparity zero and blurring out the rest of the given image. As shown in Figure 11, the left red box highlights the details of the focused area, while the right one shows the blurred background. One can observe: (i) RVR is prone to generating halo artifacts along the boundaries; (ii) SteReFo tends to generate blurred pixels at the focused area; (iii) Our method, by contrast, can keep the focused area clear and blur out the rest of the given image. To find out why RVR and SteReFo fail to generate promising predictions, we revisit their definitions and implementations. RVR is the iterative rendering without weight normalization that leads to halo artifacts along the boundaries. For SteReFo, although it can get a smooth transition of the refocus plane by assigning each pixel to multiple depth layers, such operation causes blur at the focused plane at the same time.

**Figure 12:**
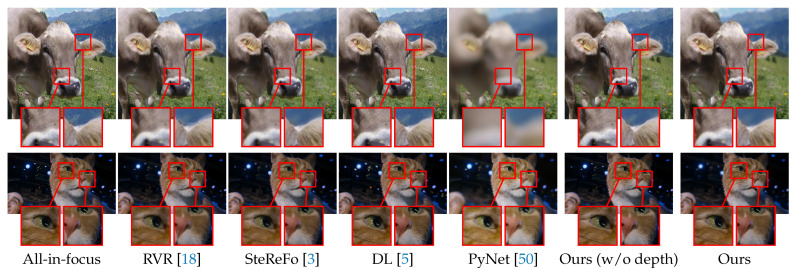
Qualitative results on the NJU2K dataset. Best viewed zoomed in on-screen.

**Figure 13:**
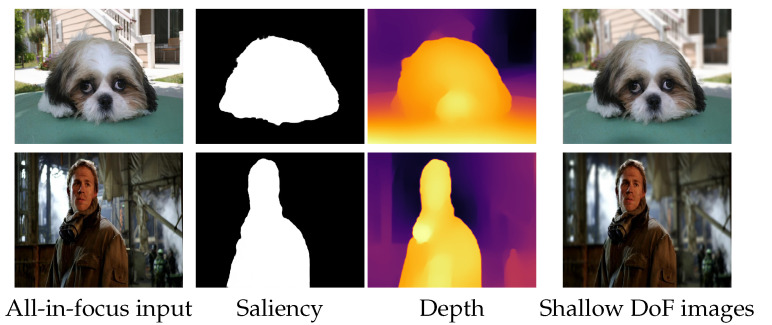
Some visual examples of our predicted saliency maps, depth maps, and shallow DoF images.

We also conduct experiments on the NJU2K [72] dataset, split into a validation set of 121 images and a test set of 364 images. We first choose the size of the blur kernel according to the performance on the validation set and then test the compared methods on the test set. Table 6 reports the PSNR and SSIM metrics of different rendering methods. The *Ours (w/o depth)* indicates a variant of our method, which blurs the background according to the salient object masks. The results imply the importance of our MDP module. Note that we did not report the performance of our method without the SOD module because our method first needs to know where to focus. The SOD module, detecting salient objects in the image, is used to determine the focal plane. In addition to the quantitative results, we also show the qualitative comparisons in Figure 12 and the visual results of different components in Figure 13. One can see: (i) RVR produces images with strong artifacts around the boundaries; (ii) SteReFo synthesizes reasonable shallow DoF images, but it tends to blur the in-focus objects; (iii) The generalization of deep learning methods is limited. For example, PyNet sometimes focuses on the background by blurring the foreground objects as it is trained on EBB [50] with no knowledge of the focal plane on NJU2K; Instead, DL can generate plausible shallow DoF images due to the use of our SOD module. (iv) The transition of boundaries is too sharp in *Ours (w/o depth)*; (v) Our predictions, by contrast, are clearer at the refocused plane and are more accurate around the boundaries. We also report the running time of our system on the NJU2K dataset. We ran our system with a NVIDIA GTX 1080Ti GPU and an E5-2650 V4 CPU for measuring the running time. The average time consuming for each image is listed as follows. The SOD module takes 0.018 s, the MDP module takes 0.121 s, and the DOF rendering module takes 0.141 s. Therefore, the whole system takes 0.28 s for rendering a shallow DoF image.

**Figure 14:**
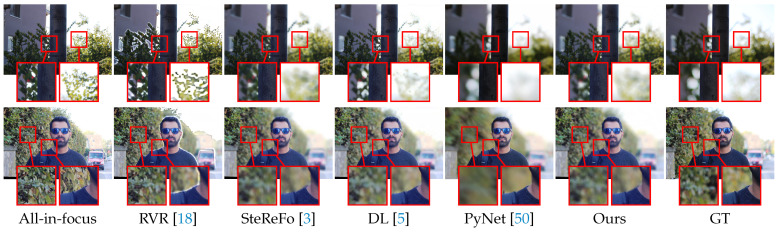
Qualitative results on the EBB dataset. Best viewed zoomed in on-screen.

To further verify the effectiveness of the overall framework, we compare with other state-of-the-art methods on the EBB [50] dataset. This dataset provides 4694 shallow/wide DoF image pairs captured by a Canon 7D DSLR with 50 mm f/1.8 lenses. We create a training set of 3694 images for training deep learning models, a validation set of 500 images for model selection, and a test set of 500 images for model evaluation. Table 7 shows the quantitative results of different methods. One can observe that our method achieves the best performance in terms of PSNR. PyNet, trained to map the narrow-aperture images into shallow DoF photos in an end-to-end manner, achieves the best SSIM result. In addition to the quantitative comparisons, we also present the qualitative results in Figure 14. In general, our method can synthesize realistic shallow DoF images on the EBB dataset.

**Figure 15:**
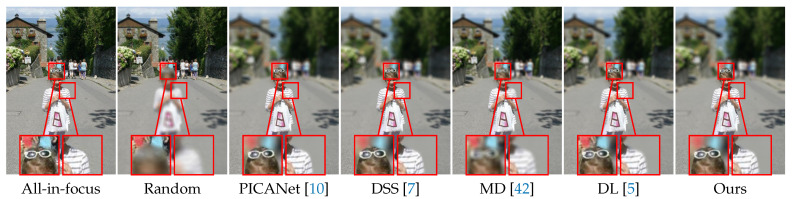
Impact of the SOD module and the MDP module. Best viewed zoomed in on-screen.

**Ablation studies:** To study the impact of the SOD module and the MDP module, we further conduct ablation studies on the NJU2K dataset. In particular, we replace our SOD module with PICANet [10], DSS [7] and a randomly selected focal plane, respectively. From Table 8, one can observe that the performance of the saliency-based methods is close. It makes sense because our current rendering method does not rely too much on the accuracy of the SOD module. We use the SOD module to guide the focal plane. Nevertheless, that does not mean the accuracy of the SOD module is not important. If its performance is too poor, the rendering results will certainly be affected (see the *Random* in Table 8). Furthermore, we replace our MDP module with other two methods (i.e., MD [42] and DL [5]) to study the impact of the MDP module. From Table 8, one can observe that with the increase of the MDP module, the rendering performance is also improved. This indicates that the more accurate the MDP model, the better the rendering results. Figure 15 demonstrates the qualitative comparisons.

## 5. Conclusions

This paper presents an automatic shallow DoF system consisting of a SOD module, an MDP module, and a DoF rendering module. The SOD module is used to determine the refocused depth, and the MDP module is used to control the degree of blur. We show that explicit modeling of the pairwise relations benefits both SOD and MDP. In particular, we propose a label-guided ranking loss for SOD. The loss comprises a heterogeneous ranking term that improves the interclass distinction and a homogeneous ranking term that enhances the intraclass consistency. To synthesize realistic shallow DoF images, we further propose an S2G method. By combing the SOD module, the MDP module, and the DoF rendering module, our system can generate realistic shallow DoF images. Besides, our method, capable of adjusting the focal plane and blur degree, is flexible in real-world applications. By changing the point spread function and the size of the blur kernel, our method can control the shape and visual quality of the defocused area. Although our system is able to generate realistic shallow DoF from an arbitrary image, it depends too much on the quality of the predicted depth. In the future, we plan to further improve the quality of monocular depth prediction.

## Figures and Tables

**Table 1:** Quantitative results of the proposed loss function with different configurations. $L_{hete}$ means only using the heterogeneous ranking loss, $L_{hete}+{\tilde{L}}_{co-bg}$ means adopting the heterogeneous ranking loss and the consistency loss of cobackground pairs, $L_{hete}+{\tilde{L}}_{co-sal}$ means adopting the heterogeneous ranking loss and the consistency loss of co-saliency pairs, and $L_{hete}+L_{homo}$ indicates utilizing the proposed label-guided ranking loss. The best performance is **boldfaced**.

Loss	DUT-O			DUTS			HKU-IS			ECSSD			PASCALS			SOD		
	$F_{\beta }↑$	MAE↓	S↑	$F_{\beta }↑$	MAE↓	S↑	$F_{\beta }↑$	MAE↓	S↑	$F_{\beta }↑$	MAE↓	S↑	$F_{\beta }↑$	MAE↓	S↑	$F_{\beta }↑$	MAE↓	S↑
$L_{hete}$	0.804	0.063	0.813	0.857	0.052	0.840	0.918	0.039	0.891	0.931	0.044	0.904	0.858	0.071	0.839	0.856	0.102	0.803
$L_{hete}+{\tilde{L}}_{co-sal}$	0.805	0.064	0.811	0.859	0.052	0.839	0.919	0.040	0.888	0.932	0.043	0.903	0.858	0.072	0.836	0.858	**0.097**	**0.808**
$L_{hete}+{\tilde{L}}_{co-bg}$	0.813	**0.055**	0.833	0.868	**0.045**	0.858	0.925	**0.035**	**0.907**	0.937	0.042	0.916	**0.864**	**0.069**	**0.851**	0.859	0.108	0.792
$L_{hete}+L_{homo}$	**0.817**	0.056	**0.834**	**0.870**	**0.045**	**0.859**	**0.926**	**0.035**	**0.907**	**0.939**	**0.040**	**0.917**	0.863	0.070	0.849	**0.863**	0.104	0.804

**Table 2:** Comparison of different loss functions on six datasets. Our loss achieves the best performance under the same setting. The best performance is **boldfaced**.

Loss	DUT-O			DUTS			HKU-IS			ECCSD			PASCALS			SOD		
	$F_{\beta }↑$	MAE↓	S↑	$F_{\beta }↑$	MAE↓	S↑	$F_{\beta }↑$	MAE↓	S↑	$F_{\beta }↑$	MAE↓	S↑	$F_{\beta }↑$	MAE↓	S↑	$F_{\beta }↑$	MAE↓	S↑
MarginRank	0.779	0.059	0.808	0.822	0.049	0.832	0.896	0.037	0.883	0.918	0.042	0.896	0.838	0.071	0.828	0.835	0.105	0.782
MAE	0.802	**0.052**	0.823	0.853	0.052	0.842	0.920	**0.032**	0.899	0.934	**0.040**	0.905	0.861	**0.065**	0.842	0.848	0.112	0.761
MSE	0.804	0.062	0.829	0.860	0.051	0.855	0.921	0.044	0.904	0.933	0.053	0.909	0.861	0.077	**0.852**	0.852	0.118	0.777
BCE	0.804	0.058	0.829	0.861	0.048	0.856	0.923	0.039	0.906	0.937	0.046	0.915	0.857	0.076	0.846	0.855	0.114	0.781
Ours	**0.817**	0.056	**0.834**	**0.870**	**0.045**	**0.859**	**0.926**	0.035	**0.907**	**0.939**	**0.040**	**0.917**	**0.864**	0.070	0.849	**0.863**	**0.104**	**0.804**

**Table 3:** Comparison of different salient object detection (SOD) methods on six datasets. The best performance is **boldfaced** and the second best is underlined. † means that the method is postprocessed by Conditional Random Field (CRF).

Methods	DUT-O			DUTS			HKU-IS			ECSSD			PASCALS			SOD		
	$F_{\beta }↑$	MAE↓	S↑	$F_{\beta }↑$	MAE↓	S↑	$F_{\beta }↑$	MAE↓	S↑	$F_{\beta }↑$	MAE↓	S↑	$F_{\beta }↑$	MAE↓	S↑	$F_{\beta }↑$	MAE↓	S↑
**VGG-16 backbone**																		
KSR	0.678	0.131	0.708	–	–	–	0.792	0.120	0.729	0.829	0.132	0.763	0.762	0.154	0.716	0.741	0.197	0.633
DCL †	0.757	0.086	0.771	–	–	–	0.907	0.055	0.877	0.901	0.075	0.868	–	–	–	–	–	–
UCF	0.730	0.120	0.760	0.772	0.112	0.777	0.888	0.062	0.875	0.903	0.069	0.883	0.848	0.115	0.806	0.805	0.148	0.763
GBR	0.759	0.073	0.806	0.774	0.073	0.798	0.891	0.057	0.877	0.909	0.066	0.887	0.821	0.107	0.807	0.819	0.130	0.762
Amulet	0.743	0.098	0.781	0.777	0.085	0.796	0.897	0.051	0.886	0.915	0.059	0.894	0.828	0.100	0.818	0.795	0.144	0.755
DSS †	0.781	0.063	0.790	–	–	–	0.916	0.040	0.878	0.921	0.052	0.882	0.831	0.093	0.799	0.843	0.122	0.746
BdMPM	0.774	0.064	0.809	0.851	0.049	0.851	0.921	0.039	0.907	0.928	0.045	0.911	0.855	0.074	0.844	0.852	0.106	0.790
PiCANet	0.794	0.068	0.826	0.851	0.054	0.851	0.921	0.042	0.906	0.931	0.047	0.914	0.856	0.078	0.848	0.850	0.101	0.793
PiCANet †	0.784	0.059	0.815	0.850	0.045	0.839	0.925	0.031	0.904	0.933	0.036	0.910	0.856	0.069	0.841	0.834	0.095	0.776
RAS	0.786	0.062	0.814	0.831	0.059	0.828	0.913	0.045	0.887	0.921	0.056	0.893	0.850	0.101	0.735	0.847	0.123	0.767
MLMSNet	0.774	0.064	0.809	0.851	0.049	0.851	0.921	0.039	0.907	0.928	0.045	0.911	**0.881**	0.074	0.794	0.852	0.106	0.790
Ours	0.787	0.063	0.808	0.853	0.049	0.845	0.917	0.039	0.897	0.922	0.052	0.895	0.850	0.076	0.836	0.841	0.120	0.771
Ours †	0.789	0.056	0.807	0.858	0.043	0.843	0.924	0.032	0.899	0.928	0.046	0.893	0.855	0.071	0.831	0.840	0.117	0.762
**Resnet-50 backbone**																		
SRM	0.769	0.069	0.798	0.827	0.059	0.824	0.906	0.046	0.887	0.917	0.054	0.895	0.838	0.084	0.834	0.840	0.126	0.745
DGRL	0.779	0.063	0.810	0.834	0.051	0.836	0.914	0.037	0.897	0.925	0.043	0.906	0.848	0.074	0.839	0.844	0.104	0.777
PiCANet	0.803	0.065	0.832	0.860	0.050	0.859	0.919	0.043	0.904	0.935	0.047	0.917	0.857	0.075	**0.854**	0.853	0.103	0.793
PiCANet †	0.804	0.054	0.826	0.866	0.040	0.849	0.927	0.031	0.905	0.940	0.035	0.916	0.859	**0.064**	0.846	0.851	**0.094**	0.780
Ours	0.809	0.057	0.827	0.866	0.046	0.856	0.925	0.036	0.905	0.936	0.042	0.913	0.862	0.071	0.846	0.853	0.103	0.791
Ours †	0.810	0.051	0.827	0.871	0.040	0.855	0.932	0.029	0.908	0.942	0.035	0.913	0.862	0.066	0.841	0.857	0.106	0.783
**DLA-60 backbone**																		
Ours	**0.817**	0.056	0.834	0.870	0.045	0.859	0.926	0.035	0.907	0.939	0.040	0.917	0.863	0.070	0.849	0.863	0.104	**0.804**
Ours †	**0.817**	**0.050**	**0.835**	**0.874**	**0.038**	**0.860**	**0.933**	**0.028**	**0.911**	**0.945**	**0.033**	**0.918**	0.863	0.065	0.845	**0.865**	0.100	0.794

**Table 4:** Cross-dataset evaluation on six RGB-D datasets. The best performance is **boldfaced**.

Methods	Ibims			TUM			Sintel			NYUDv2			KITTI			DIODE		
	Ord↓	δ>1.25↓	Rel↓	Ord↓	δ>1.25↓	Rel↓	Ord↓	δ>1.25↓	Rel↓	Ord↓	δ>1.25↓	Rel↓	Ord↓	δ>1.25↓	Rel↓	Ord↓	δ>1.25↓	Rel↓
DIW [41]	46.97	39.30	0.232	39.62	37.42	0.270	43.50	56.21	0.405	37.33	36.85	0.210	29.92	51.45	0.306	45.40	42.25	0.307
DL [5]	40.92	34.75	0.211	31.62	25.26	0.205	36.63	48.20	0.407	31.67	32.71	0.196	25.40	45.32	0.271	43.77	40.04	0.311
RW [15]	33.13	30.46	0.220	30.07	25.16	0.200	31.12	45.46	0.410	26.76	28.86	0.178	16.40	31.32	0.207	39.42	38.27	0.320
MD [42]	36.82	31.31	0.200	31.88	26.86	0.226	38.07	53.56	0.422	27.84	29.69	0.182	17.50	36.32	0.238	39.07	39.03	0.323
Y3D [44]	31.73	26.02	0.174	30.37	26.36	0.230	33.88	47.50	0.329	26.39	23.13	0.153	15.08	30.20	0.185	35.57	36.48	**0.276**
MC [43]	31.30	21.53	0.152	26.22	26.06	0.204	37.49	44.85	0.476	25.48	23.70	0.159	22.46	48.02	0.280	40.85	39.29	0.337
HRWSI [14]	27.23	23.09	0.170	**25.67**	**19.41**	**0.194**	30.70	44.84	0.402	23.21	23.50	0.157	14.01	25.40	0.179	33.11	34.44	0.301
Ours	**25.05**	**20.40**	**0.156**	25.93	20.11	0.199	**29.29**	**44.15**	**0.390**	**22.46**	**22.45**	**0.152**	**13.70**	**24.86**	**0.178**	**32.37**	**33.96**	0.310

**Table 5:** Quantitative results of different DoF rendering methods on the 4DLF dataset [70]. The best performance is **boldfaced**.

Focused Disparity	−1.5		−0.75		0		0.75		1.5		Average	
Method	PSNR	SSIM	PSNR	SSIM	PSNR	SSIM	PSNR	SSIM	PSNR	SSIM	PSNR	SSIM
RVR [18]	25.82	0.85	27.69	0.88	29.46	0.90	29.14	0.90	27.94	0.87	28.01	0.88
SteReFo [3]	33.91	0.97	35.67	0.97	37.24	0.97	36.78	0.97	35.34	0.97	35.79	0.97
S2G (Ours)	**39.56**	**0.98**	**40.51**	**0.98**	**39.47**	**0.98**	**39.71**	**0.98**	**40.87**	**0.98**	**40.03**	**0.98**

**Table 6:** Quantitative results of different rendering methods on the NJU2K [72] dataset.

	RVR [18]	SteReFo [3]	DL [5]	PyNet [50]	Ours (w/o Depth)	Ours
PSNR	30.82	32.06	29.56	24.69	31.40	**33.04**
SSIM	0.89	0.90	0.86	0.84	0.89	**0.91**

**Table 7:** Quantitative results of different methods on the EBB [50] dataset.

	RVR [18]	SteReFo [3]	DL [5]	PyNet [50]	Ours
PSNR	22.82	23.54	23.63	23.37	**23.79**
SSIM	0.82	0.85	0.86	**0.87**	0.86

**Table 8:** Ablation study on the impact of different components.

	Random	PICANet [10]	DSS [7]	MD [42]	DL [5]	Ours
PSNR	29.77	**33.04**	32.95	31.84	32.23	**33.04**
SSIM	0.85	**0.91**	**0.91**	0.89	0.90	**0.91**

## Data Availability

The datasets analyzed in this study are available through their respective respository pages. The salient object detection datasets can be found here: (SOD [56] at https://www.elderlab.yorku.ca/resources/salient-objects-dataset-sod/), (ECSSD [57] at http://www.cse.cuhk.edu.hk/leojia/projects/hsaliency/dataset.html), (PASCAL-S [58] at http://cbs.ic.gatech.edu/salobj/), (HKU-IS [24] at https://i.cs.hku.hk/~yzyu/research/deep_saliency.html), (DUT-OMRON [59] at http://saliencydetection.net/dut-omron/), and (DUTS [55] at http://saliencydetection.net/duts/). The monoculuar depth predition datasets can be found here: (HRWSI [14] at https://kexianhust.github.io/Structure-Guided-Ranking-Loss/), (NYUDv2 [64] at https://cs.nyu.edu/~silberman/datasets/nyu_depth_v2.html), (Ibims [65] at https://www.bgu.tum.de/lmf/ibims1/), (TUM [66] at https://vision.in.tum.de/data/datasets/rgbd-dataset/download), (KITTI [67] at http://www.cvlibs.net/datasets/kitti/eval_depth.php?benchmark=depth_prediction), (Sintel [68] at http://sintel.is.tue.mpg.de/downloads), and (DIODE [69] at https://diode-dataset.org/). The DoF related datasets can be found here: (4DLF [70] at https://lightfield-analysis.uni-konstanz.de/), (NJU2K [72] at https://drive.google.com/file/d/1R1O2dWr6HqpTOiDn6hZxUWTesOSJteQo/view), and (EBB [50] at http://people.ee.ethz.ch/~ihnatova/pynet-bokeh.html), all accessed on 5 March 2021.
